# Association between Rhesus Blood Groups and Malaria Infection: A Systematic Review and Meta-Analysis

**DOI:** 10.3390/tropicalmed8040190

**Published:** 2023-03-25

**Authors:** Yanisa Rattanapan, Thitinat Duangchan, Kinley Wangdi, Aongart Mahittikorn, Manas Kotepui

**Affiliations:** 1 Medical Technology, School of Allied Health Sciences, Walailak University, Nakhon Si Thammarat 80160, Thailand; 2 Hematology and Transfusion Science Research Center, Walailak University, Nakhon Si Thammarat 80160, Thailand; 3 Department of Global Health, National Centre for Epidemiology and Population Health, College of Health and Medicine, Australian National University, Canberra, ACT 2601, Australia; 4 Department of Protozoology, Faculty of Tropical Medicine, Mahidol University, Bangkok 73170, Thailand

**Keywords:** malaria, Rhesus, blood group, *Plasmodium*, Rh, meta-analysis, systematic review

## Abstract

In the literature, there was inconsistency in the risk of malaria between individuals with Rhesus blood group positive (Rh+) and negative (Rh−). The systematic review aimed to investigate the risk of malaria among participants with different Rh blood types. All observational studies that reported the occurrence of *Plasmodium* infection and investigation of the Rh blood group were searched in five databases (Scopus, EMBASE, MEDLINE, PubMed, and Ovid). Strengthening the Reporting of Observational Studies in Epidemiology was used to assess the reporting quality in the included studies. A random-effects model was used to calculate the pooled log OR and 95% confidence intervals (CIs). Database searches yielded a total of 879 articles, of which 36 were eligible for inclusion in the systematic review. The majority of the included studies (44.4%) revealed that Rh+ individuals had a lower proportion of malaria than Rh− individuals; however, the remaining studies revealed a higher or no difference in the proportion of malaria between Rh+ and Rh− individuals. Overall, with moderate heterogeneity, the pooled results showed no difference in malaria risk between patients with Rh+ and Rh− (*p* = 0.85, pooled log OR: 0.02, 95% CI: −0.20–0.25, I^2^: 65.1%, 32 studies). The current study found no link between the Rh blood group and malaria, even though there was a moderate amount of heterogeneity. Further studies using prospective designs and a definitive method for *Plasmodium* identification are needed to investigate the risk of *Plasmodium* infection in Rh+ individuals and increase the reliability and quality of these studies.

## 1. Introduction

The Rh blood group system is a blood group with a high diversity of antigens. At present, more than 50 antigens have been identified in the Rh system, with the main antigens being C, c, D, E, and e. Rh-positive (Rh+) and Rh-negative (Rh−) blood groups refer to the presence and absence of D antigen expression on the red blood cell surface, respectively [1]. Furthermore, the Rh blood group system is the most clinically important blood group after the ABO, since antigens can provoke an immune response that makes it most likely to cause hemolytic disease of the fetus and newborn (HDFN) and hemolytic transfusion reaction (HTR) [2].

There have been few studies on the relationship between the Rh blood group system and infectious diseases. Even though previous studies have reported the relationship of the Rh blood group with the novel coronavirus disease (COVID-19), parvovirus infection, leptospirosis, HIV, and hepatitis B [3,4,5,6,7,8]. The study showed that individuals with Rh+ were at higher risk of COVID-19 infection, while individuals with Rh− were at a lower risk of COVID-19 infection [3]. Although there was an association between the Rh blood group and COVID-19 infection, the association may be due to underlying, unexplored factors such as co-morbidity rather than the Rh blood group itself. For the association between blood groups and SARS-CoV-2, more large prospective studies are needed to confirm the association [3]. Another study also showed that people with Rh+ might be at a higher risk of COVID-19 infection [4]. The Rh+ was found in a significantly high number of COVID-19 patients who were admitted to the intensive care unit (ICU), while no significant relationship was found between mortality and the Rh blood group, indicating that the association might help management of COVID-19 patients [4]. In addition to COVID-19, parvovirus B19 viraemia has been reported to be associated with Rh+ donations, suggesting a protective factor in donors who lack this antigen [5]. There was a report about the increased prevalence of the severe form of leptospirosis in people with the Rh blood group, though there was no statistically significant difference between the severity of the disease and Rh [6]. The previous study also reported that children who were Rh− were at higher risk of HIV-1 infection than children who were Rh+, suggesting that Rh blood groups may be markers for genetic susceptibility to vertical HIV infection [7]. Furthermore, Rh+ reflects the susceptibility of populations carrying hepatitis B [8].

*Plasmodium falciparum*, *Plasmodium ovale curtisi*, *Plasmodium ovale wallikeri*, *Plasmodium malariae*, *Plasmodium vivax*, and *Plasmodium knowlesi* are six species of *Plasmodium* that can infect humans and cause malaria [9]. Previous studies found an association between the ABO blood groups and *P. falciparum*, and people with A, B, and AB blood groups had an increased risk of *Plasmodium* infection than those with an O blood group [10,11,12]. In addition, people with the A and B blood groups had an increased risk of having severe malaria than those with an O blood group [13,14]. Despite several studies investigating the relationship between the Rh blood group and malaria risk, the findings between malaria risk and the Rh blood group were inconsistent in the literature. This systematic review set out to compare the likelihood of malaria infection between people with Rh+ and those with Rh− blood types.

## 2. Materials and Methods

The protocol guiding this review has been previously registered in PROSPERO (ID: CRD42023392462). The results of the systematic review were presented according to the Preferred Reporting Items for Systematic Reviews and Meta-Analyses (PRISMA) guidelines [15]. The systematic review question was developed according to the Population, Exposure, Outcome (PEO) framework to determine the association between particular risk factors and outcomes [16]. Population: patients with and without malaria. Exposure: Rh blood group (Rh+ or Rh−). Outcome: malaria infection.

### 2.1. Search Strategy

Five databases (Scopus, EMBASE, MEDLINE, PubMed, and Ovid) were explored to access articles (Table S1). A methodical search approach including MeSH words and Boolean operators was implemented: [(“Blood group” or blood-group or “blood type” or “blood antigen”) and (Rh-Hr or “Rh Hr” or Rhesus or Rh or “Rh Factors” or “Rh Factor” or “Antigen D”) and (malaria or *Plasmodium* or “Remittent Fever” or “Marsh Fever” or Paludism)] (Table S1). This search strategy was slightly different from one database to another. In addition, a manual Google Scholar search was performed to access additional articles that were not indexed in the main databases. The search was not limited to the English language or the publication year of the articles.

### 2.2. Selection of Studies and Data Extraction

All observational studies that reported the occurrence of *Plasmodium* infection using microscopic, serological, or molecular methods for the identification of *Plasmodium* species were eligible for this review. Furthermore, the Rh blood group of participants with and not without *Plasmodium* infection must be reported in the eligible studies. The Rh blood group could be determined by a slide or tube agglutination method. Articles with no clear evidence on the Rh blood group or *Plasmodium* spp. were excluded from the review. Furthermore, the following articles were excluded: malaria cases without Rh blood group assessment; full texts were unavailable or could not be accessed; reviews; data of the Rh blood group or *Plasmodium* infection could not be extracted; no data of an association between malaria and the Rh blood group; a poster; a letter to the editor; a case report; a book; and a book chapter. 

A standard extraction from a Microsoft Excel (Microsoft Corporation, Redmond, WA, USA) spreadsheet was used to import the data of the first author, publication year, time period for the study, continent, country, study design, type and number of participants, age group of participants, a diagnostic method for *Plasmodium* infection, *Plasmodium* spp., the clinical status of malaria (asymptomatic, uncomplicated, or severe malaria), number of participants with malaria (and Rh+), number of participants without malaria (and Rh+), and number of participants without malaria (and Rh−). The selection of eligible studies and extraction of data files were independently performed by the three authors (YR, MK, and TD) and were further evaluated for discrepancies by another author (AM).

### 2.3. Quality Assessment

Strengthening the Reporting of Observational Studies in Epidemiology was used to assess the reporting quality of observational studies, including cross-sectional studies, cohort studies, and case-control studies [17]. The criteria for determining the reporting quality were based on 22 checklist items. The checklist assessed the reporting quality of the study in the following parts of each study: title and abstract, introduction, methods, results, discussion, and other information. A score of 1 was awarded for each checklist item described in the articles, and a score of 0 otherwise. A summary score percentile between >75, 50–75, and <50 indicates high, moderate, and poor reporting quality of the study, respectively (see results in Table S2).

### 2.4. Data Analysis

A random-effects model has been employed to compute the combined log odds ratio (OR) and 95% confidence intervals (CIs) [18]. Under the random-effects model, the influence of larger studies on the total effect is minimal, while the influence of smaller studies is substantial [19]. The heterogeneity of outcomes between studies was determined using the inconsistency index (I^2^) of 25%, 50%, and 75% indicating small, moderate, and high degrees of heterogeneity, respectively [20]. Candidate sources of heterogeneity were determined using metaregression, and if the results of the metaregression were significant (*p* < 0.05), subsequently subgroup analysis was carried out. Factors for metaregression and subgroup analysis were publication year, design of the study, location, subjects, age range, *Plasmodium* spp., clinical status, the technique of *Plasmodium* detection, and publication quality. The fixed effect model was utilized in the sensitivity analysis to calculate the combined log OR and 95% CIs in the absence of heterogeneity. The leave-one-out meta-analysis was performed to investigate whether the result of an individual would affect the pooled estimate. Egger’s test was used to identify the effects of small studies once publication bias was detected through thorough observation of a funnel plot’s asymmetry. All statistical tests were carried out using Stata 17.0 software (StataCorp LLC, College Station, TX, USA), and statistically significant was defined as a *p* value less than 0.05.

## 3. Results

### 3.1. Search Results and Characteristics of the Included Studies

**Figure 1:**
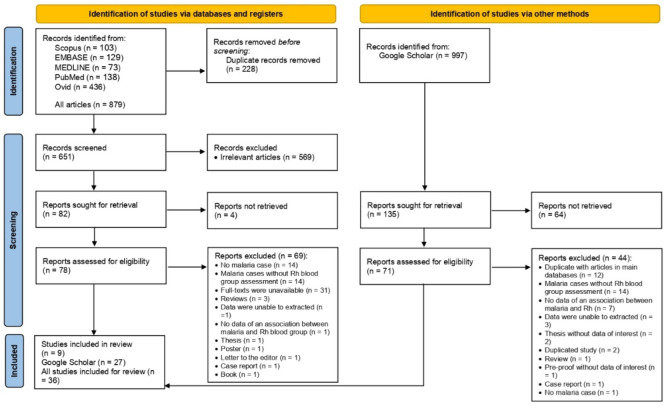
Flow diagram of study selection.

A total of 879 articles were yielded from database searching, including Scopus (n = 103), EMBASE (n = 129), MEDLINE (n = 73), PubMed (n = 138), and Ovid (n = 436). In addition, 997 articles were retrieved from searching Google Scholar. After the selection of studies, 36 studies were eligible for inclusion in the systematic review [21,22,23,24,25,26,27,28,29,30,31,32,33,34,35,36,37,38,39,40,41,42,43,44,45,46,47,48,49,50,51,52,53,54,55,56] (Figure 1). 

Table 1 displays the broad characteristics of the selected publications. Briefly, cross-sectional designs were most common among the reviewed publications (80.6%, 29) and were published between 2010 and 2019 (63.9%, 23). Nearly half (47.2%, 17) of the publications were reported from Nigeria, and the most common publications were from the enrolled blood donors (40.6%, 13). More than half (55.6%, 20) of the publications were from adult age groups. Almost one-third (27.8%, 10) of the study participants were infected with *P. falciparum*, while 66.7% (24) were asymptomatic *Plasmodium*-infected patients. The most common diagnostic test was microscopy (63.9%, 23) followed by RDT (8.3%, 3) (Table 1).

### 3.2. Reporting Quality of the Included Studies and Qualitative Synthesis

Based on the STROBE criteria, two-thirds of studies (75%) were of moderate quality [22,24,26,27,28,29,30,31,33,34,35,36,38,39,40,41,43,45,46,47,48,50,52,53,54,55]. Meanwhile, eight studies were of high quality (22.2%) [23,25,31,37,42,44,51,56], and one was of low quality (2.78%) [49] (Table S3). The systematic review included all studies, and the varied reporting quality was addressed in the metaregression analysis to investigate whether the different reporting qualities of the included studies affected the combined outcome.

For qualitative synthesis, 13 studies (36.1%) showed a higher proportion of malaria in Rh+ individuals than in Rh− individuals [21,25,27,32,38,41,42,43,46,50,53,54,56]. Among these studies, a significantly higher proportion of malaria in Rh+ individuals than in Rh− individuals was reported by three studies [25,27,41]. Meanwhile, no statistically significant difference in the proportion of malaria between Rh+ and Rh− was reported in three studies [42,46,56]. The remaining seven studies reported a higher proportion of malaria in Rh+ individuals than in Rh− individuals, though no statistical significance or statistical analysis has been performed [21,32,38,43,50,53,54]. Sixteen studies (44.4%) showed a lower proportion of malaria in Rh+ individuals than in Rh− individuals [23,24,26,29,30,31,34,37,39,40,44,45,47,48,52,55]. Among these studies, a significantly lower proportion of malaria among Rh+ individuals was reported by two studies [39,44]. Three studies reported a lower proportion of malaria among Rh+ individuals than in Rh− individuals, though no statistical significance [37,45,48]. Eleven studies reported a lower proportion of malaria among Rh+ individuals than in Rh− individuals, however, no statistical analysis has been performed [23,24,26,29,30,31,34,40,47,52,55]. The other seven studies reported the association between the Rh blood group and malaria infection [22,28,49], parasite density [33,36], or malaria severity [35,51]. No significant association between malaria and the Rh blood group was reported by three studies [22,28,49]. No association between the Rh blood group and malaria density was reported by two studies [33,36], though the study by Elnaim et al. did not perform a statistical analysis [33]. No significant association between Rh and malaria severity was reported by two studies [35,51]. 

### 3.3. Association between Rhesus Blood Groups and Malaria

**Figure 2:**
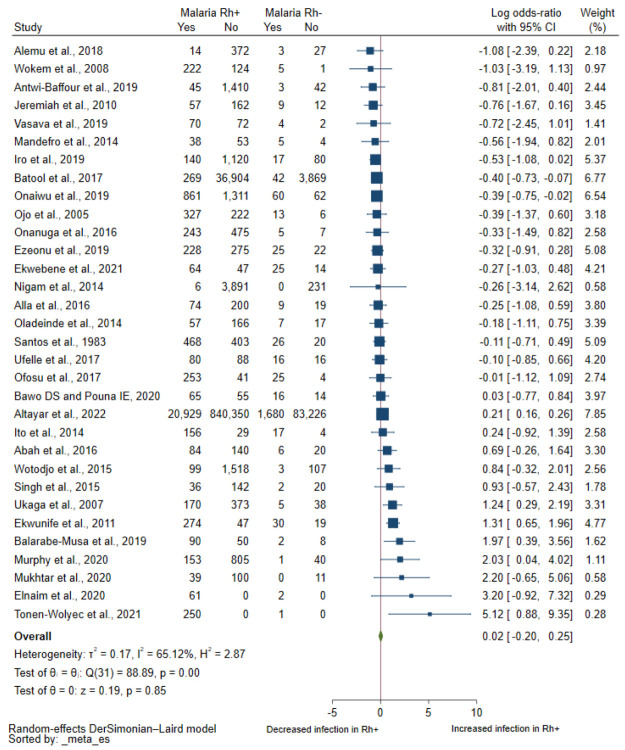
Forest plot demonstrating the odds of malaria among individuals with Rh+ and Rh− when a random effects model has been used. Abbreviations: OR, OR; CI, confidence interval. [21,23,24,25,26,27,29,30,31,32,33,34,37,38,39,40,41,42,43,44,45,46,47,48,49,50,51,52,53,54,55,56].

Thirty-two studies with a study population of 1,007,763 reported the quantitative number of participants with malaria infection and Rh blood groups, which were included in the meta-analysis of the pooled log OR [21,23,24,25,26,27,29,30,31,32,33,34,37,38,39,40,41,42,43,44,45,46,47,48,49,50,51,52,53,54,55,56]. Results of individual studies showed increased odds of having malaria among Rh+ individuals in six studies [25,27,32,42,51,53]. Meanwhile, there were decreased odds of having malaria among Rh+ individuals in two studies [29,47]. In 28 studies [21,22,23,24,26,28,30,31,33,34,35,36,37,38,39,40,41,43,44,45,46,48,49,50,52,54,55,56], there was no difference in the odds of having malaria between patients with Rh positivity and Rh negativity. Overall, the meta-analysis found no difference in the odds of having malaria between the Rh+ and Rh− patients (*p* = 0.85, pooled log OR: 0.02, 95% CI: −0.20–0.25, I^2^: 65.12%, 32 studies, Figure 2).

The metaregression analysis of covariates including publication year, design of the study, location, subjects, age range, *Plasmodium* spp., clinical status, the technique of *Plasmodium* detection, and publication quality showed no significance of these covariates on the pooled log OR (*p* > 0.05, Table 2). Therefore, the subgroup analysis of these covariates was not performed.

### 3.4. Sensitivity Analysis

**Figure 3:**
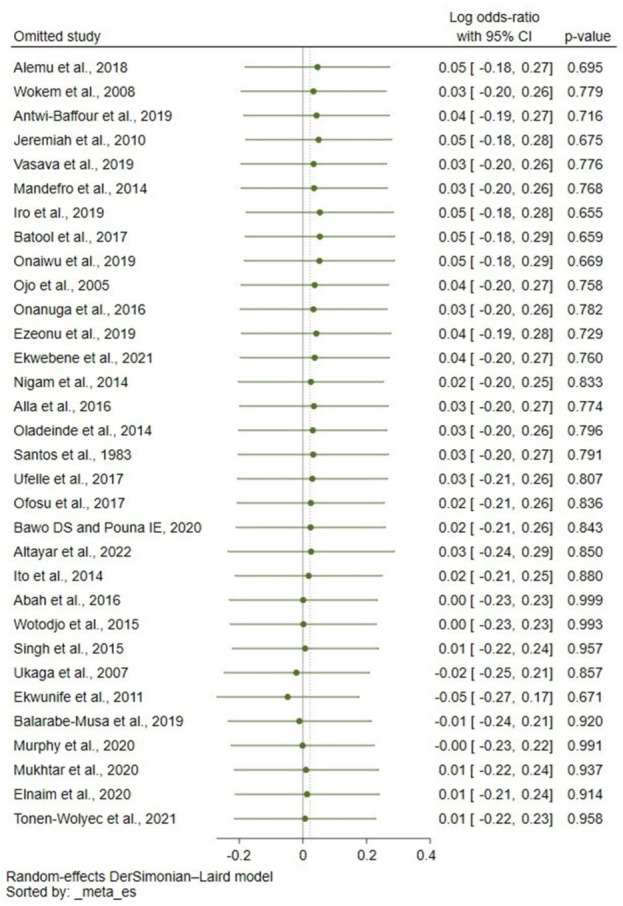
Sensitivity analysis demonstrating no individual study that affects meta-analysis results. Abbreviations: CI, confidence interval. [21,23,24,25,26,27,29,30,31,32,33,34,37,38,39,40,41,42,43,44,45,46,47,48,49,50,51,52,53,54,55,56].

When the fixed effects model was used to pool the log OR, there was a strong link between the Rh blood groups and malaria (*p* < 0.01, pooled log OR: 0.17, 95% CI: 0.13–0.22, I^2^: 65.12, 32 studies, Supplementary Figure S1). The leave-one-out meta-analysis revealed that no study was an outlier that could affect the pooled estimate, with *p* values greater than 0.05 in all rerun meta-analyses (Figure 3).

### 3.5. Publication Bias

**Figure 4:**
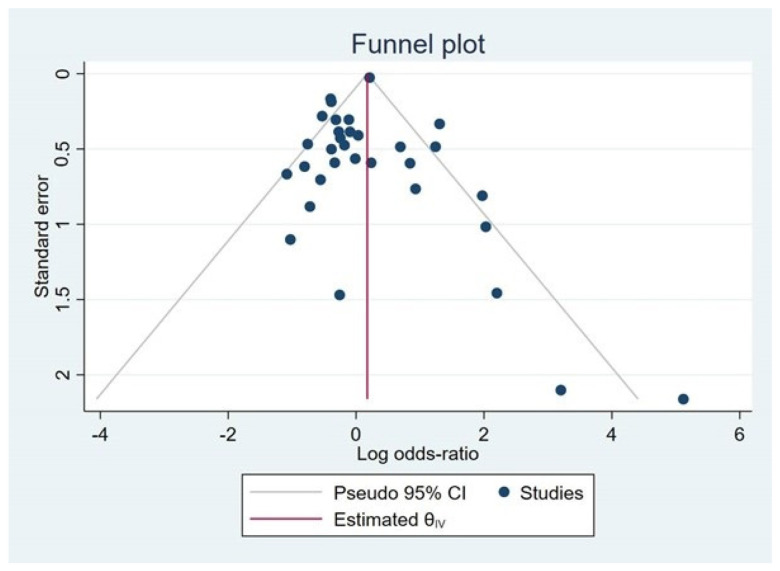
The funnel plot illustrated the unequal distribution of effect estimates on the graph. Abbreviations: CI, confidence interval; IV, inverse variance.

The funnel plot was asymmetric (Figure 4), and the significant small-study effects were found by Egger’s test (*p* = 0.02), indicating some studies were missing from the meta-analysis and causing funnel plot asymmetry. The trim and fill method showed a significant association between Rh blood groups and malaria (pooled log OR: 0.175, 95% CI: 0.13–0.22).

## 4. Discussion

Although there was inconsistency in the risk of *Plasmodium* infections between individuals with Rh+ and Rh−, the meta-analysis demonstrated no difference in the risk of *Plasmodium* infections among the two blood groups. It was true that most of the studies (44.4%) reporting an association between the Rh blood group and malaria showed a lower proportion of malaria in Rh+ individuals than in Rh− individuals, but only a few studies investigated the association using statistical analysis. In addition, several studies investigated the association between the risk of *Plasmodium* infections among individuals with Rh+ and Rh− in a small number of participants. Therefore, the variation in sample size between studies might affect the conclusion made by the random effects model, as smaller studies had more influence on the conclusion of the meta-analysis finding. Although the largest study by Altayar et al. [25], with a total number of participants of 946,185, showed a positive association between Rh+ and *Plasmodium* infection, the weight of the study was 7.85% since large studies lose influence and small studies gained influence in the random-effects model [19]. Notably, Altayar et al. [25] used the serology method, which is not a definitive method for identifying *Plasmodium* infection, thereby limiting the reliability and quality of the results presented in their study. There were different results of meta-analysis between the random effects model and the fixed effects model. The random effects model revealed no association between the Rh blood group and *Plasmodium* infection. However, the fixed effects model by combining the effect estimates in the sensitivity analysis showed a significant association between Rh+ and *Plasmodium* infection. Since each study included in the meta-analysis provided different effect sizes (the true effect sizes for all studies were not identical) and there was heterogeneity between studies, the random-effects model rather than the fixed-effect model was the more appropriate statistical model for combining effect estimates [19].

The mechanism by which the Rh blood group could serve as a protective or risk factor for malaria is unknown. The Rh blood group might alter the adhesion properties of the parasites to the RBC’s membrane and sequester itself in less desirable locations for *Plasmodium* infection. In other well-studied associations between malaria and blood group, such as the ABO blood group, individuals with blood group O had less rosetting than those with the A, B, and AB blood groups, which reduced the risk of malaria infection and/or severity due to rosetting was associated with malaria severity [14,57,58]. Blood group heterogeneity could be related to parasite adhesion and erythrocyte infection. Parasites directly penetrate red blood cells (RBCs) through the receptors on the surfaces of the blood groups. FY*1 and FY*2 are codominant alleles encoding the Duffy blood group antigens Fy^a^ and Fy^b^. These antigens give rise to four major Duffy phenotypes: Fy(a+b+), Fy(a+b−), Fy(a−b+), and Fy(a−b−) [59]. Fy(a−b−), results from a mutation in the GATA-1 of the promoter region of the *DARC* gene [60]. Duffy-glycoprotein is a receptor for the *P. vivax* parasites. Mutations in the coding region of the *FY* alleles (Duffy null phenotype) result in the absence of the receptor from RBCs and resistance to *P. vivax* infection. MN blood group antigens carried on glycophorin A (GPA), act as receptors for pathological *P. falciparum* [61]. The O blood group was more sensitive to hemolysis caused by *P. falciparum* than other blood groups. The pathogenesis is mainly dependent on the adherence of *P. falciparum* parasitized erythrocytes to the endothelium of blood vessels [62]. The A and B blood groups have been suggested to play a crucial role in cytoadherence [63]. Cytoadherence, rosetting, and sequestration are reduced in the O blood group due to the absence of A and B antigens on the surface of the blood group [64]. The O blood group may prevent patients from developing severe malaria through the reduction of rosette formation in RBCs. Rosette frequencies in parasite isolates from patients with blood group O were found to be considerably lower. This may explain why the O blood group is protective against severe malaria compared to the A, B, and AB blood groups [14]. The Knops (KN) blood group system comprises nine antigens: Kn^a^/Kn^b^ (KN1/KN2), McC^a^/McC^b^ (KN3/KN6), Sl1/Sl2 (KN4/KN7), Yka (KN5), Sl3 (KN8), and KCAM (KN9) antigens [65,66]. These antigens reside on the complement receptor one (CR1) molecule. CR1 is an RBC transmembrane glycoprotein that is responsible for regulating complement-activating enzymes and transferring immune complexes bound with activated complement components (C3b/C4b) [67]. Helgeson RBCs, which are a null variant of the Knops system, exhibited dramatically reduced rosetting, indicating that CR1-suppressed rosetting plays a role in malaria [68].

The current study contained several limitations. First, the moderate heterogeneity in the meta-analysis results may have an impact on the study’s conclusion. Second, the meta-regression analysis was used to investigate the source of the outcome’s heterogeneity. However, no probable source of heterogeneity was discovered. Third, there was a publication bias among the studies included in the meta-analysis, as visualized in the funnel plot and the results of Egger’s test, which might affect the conclusion made by this study. Fourth, the majority of the studies included in the systematic review were of moderate reporting quality, so the meta-analysis results were based on quality levels. There was no significant effect of study quality on the pooled effect estimate when study quality was included in the meta-regression analysis.

## 5. Conclusions

The current study found no link between the Rh blood group and malaria, even though there was a moderate amount of heterogeneity. Further studies using prospective designs and a definitive method for *Plasmodium* identification are needed to investigate the risk of *Plasmodium* infection in Rh+ individuals and increase the reliability and quality of these studies.

## Figures and Tables

**Table 1:** Summary characteristics of 36 studies included in the review.

Characteristics	Subgroups	Frequency (n)	Percentage (%)
Publication years	Before 2000	1	2.78
	2000–2009	4	11.1
	2010–2019	23	63.9
	2020–2022	8	22.2
Study design	Cross-sectional studies	29	80.6
	Case-control studies	6	16.7
	Not specified	1	2.78
Country	Nigeria	17	47.2
	Congo	1	2.78
	Cameroon	1	2.78
	Uganda	1	2.78
	Sudan	1	2.78
	Spain	1	2.78
	Niger	1	2.78
	Ghana	1	2.78
	Ethiopia	1	2.78
	Brazil	1	2.78
	Colombia	1	2.78
	India	1	2.78
	Saudi Arabia	1	2.78
	Pakistan	1	2.78
Participants	Blood donors	13	40.6
	Patients with malaria	5	36.1
	Malaria suspected patients	4	11.1
	Participants in community	4	11.1
	Patients in the hospital	4	11.1
	Pregnant women	2	5.56
	University’s staff	1	2.78
	University’s students	1	2.78
	Pregnant and nonpregnant women	1	2.78
	Students	1	2.78
*Plasmodium* spp.	*P. falciparum*	10	27.8
	*P. falciparum*, *P. vivax*	5	13.9
	*P. falciparium*, *P. malariae*	2	5.56
	Not specified	19	52.8
Clinical status of malaria	Asymptomatic malaria	24	66.7
	Uncomplicated malaria	8	22.2
	Asymptomatic and uncomplicated malaria	2	5.56
	Uncomplicated and severe malaria	2	5.56
Age groups	Adult	20	55.6
	All age ranges	8	22.2
	Children	4	11.1
	Not specified	4	11.1
Method for malaria detection	Microscopy	23	63.9
	RDT	3	8.33
	Microscopy, RDT	2	5.56
	Serology	2	5.56
	Microscopy/RDT/Molecular method	1	2.78
	Molecular method	2	5.56
	Not specified	3	8.33

Abbreviations: RDT, rapid diagnostic test.

**Table 2:** Metaregression results.

Covariates	*p* Value	I-Squared Residual (%)	tau2	R-Squared (%)	Number of Studies
Publication year	0.51	58.13	0.24	0	32
Study design	0.47	64.02	0.21	0	31
Continent	0.76	64.11	0.26	0	32
Country	0.27	58.99	0.31	0	32
Participants	0.71	70.8	0.26	0	32
*Plasmodium* spp.	0.86	74.52	0.86	0	14
Clinical status	0.21	66.37	0.19	0	32
Age group	0.13	67.48	0.18	0	28
Method for malaria detection	0.48	60.98	0.35	0	30
Reporting quality of the included studies	0.96	61.52	0.26	0	32

## Data Availability

Data is contained within the article or supplementary material.

## Supplementary Materials

The following supporting information can be downloaded at: https://www.mdpi.com/article/10.3390/tropicalmed8040190/s1, Table S1: Search terms; Table S2: Details of the included studies; Table S3: Quality of the included studies. Figure S1. Forest plot demonstrating the odds of malaria among individuals with Rh+ and Rh− when a fixed effects model has been used. Abbreviations: OR, OR; CI, confidence interval.
