# Supplementary material for: Association between Rhesus Blood Groups and Malaria Infection: A Systematic Review and Meta-Analysis
Source: Trop Med Infect Dis. 2023 Mar 25;8(4):190. doi: 10.3390/tropicalmed8040190 (PMC10145489; doi:10.3390/tropicalmed8040190)
Supplement: Supplementary file 1 [file tropicalmed-08-00190-s001.zip › Table S1. Search terms.pdf]

Table S1. Search strategy

PubMed

6 December 2022

No.	Query	Results
4	#1 AND #2 AND #3	138
3	malaria OR Plasmodium OR "Remittent Fever" OR "Marsh Fever" OR Paludism	120,137
2	Rh-Hr OR "Rh Hr" OR Rhesus OR Rh OR "Rh Factors" OR "Rh Factor" OR "Antigen D"	637,634
1	"Blood group" OR blood-group OR "blood type" OR "blood antigen"	723,750

Scopus

6 December 2022

No.	Query	Results
4	1 AND 2 AND 3	103
3	TITLE-ABS-KEY (malaria OR plasmodium OR "remittent fever" OR "marsh fever" OR paludism)	153,150
2	TITLE-ABS-KEY (rh-hr OR "rh hr" OR rhesus OR rh OR "rh factors" OR "rh factor" OR "antigen d")	181,691
1	TITLE-ABS-KEY ("blood group" OR blood-group OR "blood type" OR "blood antigen")	66,949

MEDLINE

No.	Search terms/Search strategy	Date
1	("Blood group" OR blood-group OR "blood type" OR "blood antigen") AND (Rh-Hr OR "Rh Hr" OR Rhesus OR Rh OR "Rh Factors" OR "Rh Factor" OR "Antigen D") AND (malaria OR Plasmodium OR "Remittent Fever" OR "Marsh Fever" OR Paludism)<br><br>Search results: 73	**6 December 2022**

Embase

No.	Search terms/Search strategy	Date

1	(malaria OR plasmodium OR 'remittent fever' OR 'marsh fever' OR paludism) AND ('rh hr' OR rhesus OR rh OR 'rh factors' OR 'rh factor' OR 'antigen d') AND ('blood group'/exp OR 'blood group' OR 'blood type' OR 'blood antigen'/exp OR 'blood antigen')<br><br>Search results: 129	**6 December 2022**

Ovid

**No.**	**Search terms/Search strategy**	**Date**
1	("Blood group" OR blood-group OR "blood type" OR "blood antigen") AND (Rh-Hr OR "Rh Hr" OR Rhesus OR Rh OR "Rh Factors" OR "Rh Factor" OR "Antigen D") AND (malaria OR Plasmodium OR "Remittent Fever" OR "Marsh Fever" OR Paludism)<br><br>Filter: limit to articles with abstracts<br>Search results: 436	**6 December 2022**
