# Supplementary figures and images for: Association between Rhesus Blood Groups and Malaria Infection: A Systematic Review and Meta-Analysis
Source: Trop Med Infect Dis. 2023 Mar 25;8(4):190. doi: 10.3390/tropicalmed8040190 (PMC10145489; doi:10.3390/tropicalmed8040190)

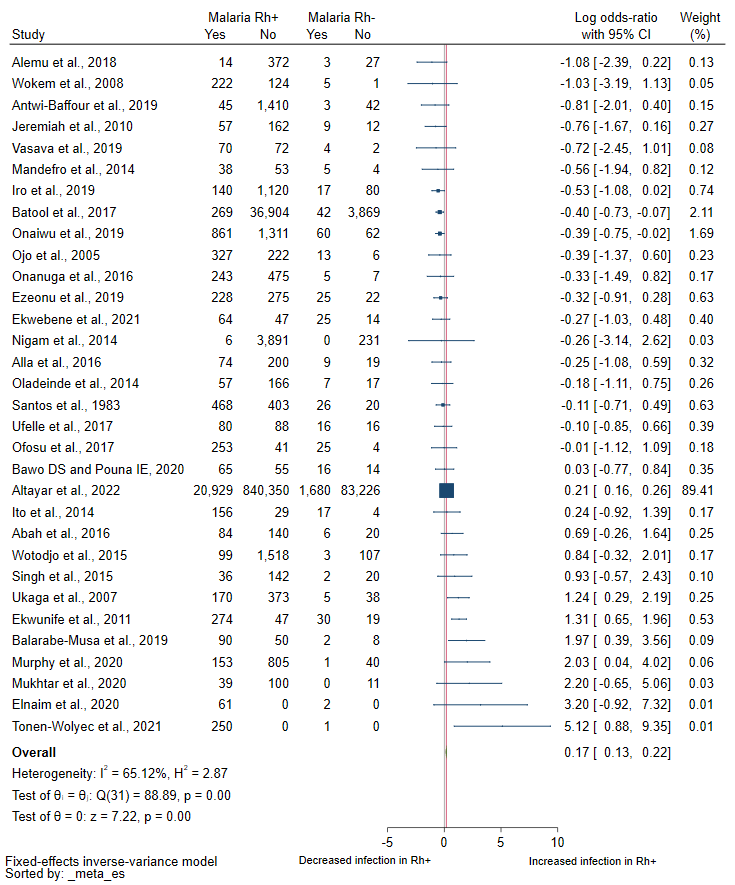

Supplement: Supplementary file 1 [file tropicalmed-08-00190-s001.zip › Supplementary Figure S1. Fixed effects model.tif]
